# Protocol for the electroencephalography guidance of anesthesia to alleviate geriatric syndromes (ENGAGES-Canada) study: A pragmatic, randomized clinical trial

**DOI:** 10.12688/f1000research.19213.2

**Published:** 2023-01-13

**Authors:** Alain Deschamps, Tarit Saha, Renée El-Gabalawy, Eric Jacobsohn, Charles Overbeek, Jennifer Palermo, Sophie Robichaud, Andrea Alicia Dumont, George Djaiani, Jo Carroll, Morvarid S. Kavosh, Rob Tanzola, Eva M. Schmitt, Sharon K. Inouye, Jordan Oberhaus, Angela Mickle, Arbi Ben Abdallah, Michael S. Avidan, Canadian Perioperative Anesthesia Clinical Trials Group

**Affiliations:** 1 Department of Anesthesiology and Pain Medicine, Montreal Heart Institute and Universite de Montreal, Montreal, Quebec, H1T 1C8, Canada; 2 Department of Anesthesiology and Perioperative Medicine, Queen's University, Kingston, Kingston, Ontario, Canada; 3 Department of Clinical Health Psychology, Anesthesiology, Perioperative and Pain Medicine, University of Manitoba, Winnipeg, Manitoba, Canada; 4 Departments of Anesthesia and Internal Medicine, University of Manitoba, Winnipeg, Manitoba, Canada; 5 Department of Anesthesiology and Pain Medicine, University of Montreal, Montreal, Quebec, Canada; 6 Montreal Heart Institute, Montreal, Quebec, H1T 1C8, Canada; 7 Montreal Health Innovation Coordinating Center, Montreal Heart Institute, Montreal, Quebec, Canada; 8 Department of Anesthesia, University of Toronto, Toronto, Ontario, Canada; 9 Department of Anesthesiology, Perioperative and Pain Medicine, Max Rady College of Medicine, University of Manitoba, Winnipeg, Manitoba, Canada; 10 Department of Medicine, Beth Israel Deaconess Medical Center, Boston, Massachussetts, USA; 11 Department of Anesthesiology, Washington University School of Medicine, St-Louis, Missouri, USA; 12 Department of Anesthesia, University of Manitoba, Winnipeg, Manitoba, Canada

**Keywords:** EEG suppression, geriatric outcomes, postoperative delirium, cardiac surgery, anesthetic management, cardiopulmonary bypass, volatile anesthetics, perioperative risk factors

## Abstract

**Background:**  There is some evidence that electroencephalography guidance of general anesthesia can decrease postoperative delirium after non-cardiac surgery.  There is limited evidence in this regard for cardiac surgery.  A suppressed electroencephalogram pattern, occurring with deep anesthesia, is associated with increased incidence of postoperative delirium (POD) and death.  However, it is not yet clear whether this electroencephalographic pattern reflects an underlying vulnerability associated with increased incidence of delirium and mortality, or whether it is a modifiable risk factor for these adverse outcomes.

**Methods:**  The **E**lectroe **n**cephalography **G**uidance of **A**nesthesia to Alleviate **Ge**riatric **S**yndromes ( **ENGAGES-Canada**) is an ongoing pragmatic 1200 patient trial at four Canadian sites.  The study compares the effect of two anesthetic management approaches on the incidence of POD after cardiac surgery.  One approach is based on current standard anesthetic practice and the other on electroencephalography guidance to reduce POD. In the guided arm, clinicians are encouraged to decrease anesthetic administration, primarily if there is electroencephalogram suppression and secondarily if the EEG index is lower than the manufacturers recommended value (bispectral index (BIS) or WAVcns below 40 or Patient State Index below 25).  The aim in the guided group is to administer the minimum concentration of anesthetic considered safe for individual patients.  The primary outcome of the study is the incidence of POD, detected using the confusion assessment method or the confusion assessment method for the intensive care unit; coupled with structured delirium chart review.  Secondary outcomes include unexpected intraoperative movement, awareness, length of intensive care unit and hospital stay, delirium severity and duration, quality of life, falls, and predictors and outcomes of perioperative distress and dissociation.

**Discussion:**  The ENGAGES-Canada trial will help to clarify whether or not using the electroencephalogram to guide anesthetic administration during cardiac surgery decreases the incidence, severity, and duration of POD.

**Registration: **ClinicalTrials.gov ( NCT02692300) 26/02/2016

## Background

### Delirium and cardiac surgery

Postoperative delirium (POD) is an acute and typically reversible syndrome, characterized by a fluctuating level of consciousness and disturbances in attention and cognition ^1^. Patients undergoing cardiac surgery are at especially high risk of developing POD ^2– 9^. The incidence of POD after cardiac surgery is estimated to be between 20% and 70% depending on the population studied and the methods used to assess delirium ^2– 9^. Delirium has been described either as hyperactive or hypoactive, the latter being more difficult to diagnose in the clinical setting than the former ^1^. As one of the most common complications after cardiac surgery in older adults, POD is associated with prolonged length of hospital and intensive care unit (ICU) stay, increased morbidity and mortality, functional and cognitive decline, and is often associated with placement in long-term care facility ^6, 7, 10^.

Several non-modifiable risk factors at the time of surgery are associated with POD including age 60 years and older, preexisting cognitive impairment, psychiatric comorbidities, and low baseline regional cerebral oxygen saturation ^8, 9, 11– 13^. Possible precipitating risk factors specific to cardiac surgery include the type of cardiac surgery, cardiopulmonary bypass time, and the number of blood product transfusions ^7, 9^. The identification of potentially modifiable perioperative risk factors, for example relating to the conduct of general anesthesia for cardiac surgery ^7^, could positively impact this major health issue.

### Electroencephalogram guided anesthetic management and delirium

While the use of processed electroencephalogram (EEG) indices to monitor depth of anesthesia is relatively prevalent in the anesthesiology community, these have mainly been directed towards avoiding patient awareness during surgery ^14– 16^. Recent interest has shifted to the potential for intraoperative EEG monitoring to prevent postoperative delirium ^17, 18^. The notion is that there might be specific EEG patterns that are strongly associated with POD, and these can be avoided through alterations in anesthetic management. One of these patterns is burst suppression, which is characterized by an isoelectric or suppressed EEG pattern followed by a short burst of high amplitude activity. Burst suppression is considered to be “a strong synchronized outflow of thalamic discharges to a widely unresponsive cortex” ^19^. Burst suppression is not encountered in normal physiological conditions such as sleep, but is associated with coma, induced hypothermia, some forms of epilepsy, cerebral hypoxia, and deep general anesthesia ^20^. Both the occurrence of burst suppression and the cumulative duration of EEG suppression during general anesthesia have been associated with POD, but without clear evidence for causality ^3, 21, 22^. The risk for burst suppression during general anesthesia might be increased by older age, medical comorbidities, a greater intraoperative dose of benzodiazepines, and, most importantly, increased intraoperative volatile or intravenous anesthetic concentration ^3, 21^. The combination of EEG suppression and hypotension has been associated with increased mortality at 90 days post-surgery ^23^. We therefore hypothesized that intraoperative guidance of anesthesia with the aim of avoiding or minimizing EEG suppression could decrease the incidence of POD in cardiac surgery patients. Evidence for this hypothesis is bolstered by recently published meta-analyses of randomized trials, which reported that using a processed EEG monitor to guide anesthetic administration is likely to substantially decrease the incidence of POD ^7, 24, 25^. While a recent randomized controlled trial using a strategy to avoid EEG suppression in older adults undergoing major surgery was not shown to be effective in decreasing the incidence of POD ^26^, this strategy has not been tested specifically in older cardiac surgery patients, a population with many risk factors and a high incidence of POD.

### The ENGAGES-Canada study: potential impact

The intent of the ENGAGES-Canada study is to determine if EEG-guided anesthesia to reduce anesthetic agent administration, thereby minimizing episodes and durations of EEG suppression, can effectively decrease the incidence of POD and its downstream sequelae in patients undergoing cardiac surgery ( Table 1). The approach of minimizing periods of EEG suppression specifically during cardiac anesthesia has not been previously tested in a large clinical trial. Recently published meta-analyses have suggested that EEG guidance of anesthesia decreases the incidence of POD by more than a third ^25^. If this result is reproduced in subsequent rigorous clinical trials, it will have important implications for how anesthesia is administered – especially to older adults. On the other hand, premature implementation based on the existing preliminary evidence might have unintended negative consequences. It is within this context that the ENGAGES trial and ENGAGES-Canada will provide critically clarifying information ^27, 28^. The ENGAGES trial addressed the question in unselected older adults undergoing major surgery ^26^. ENGAGES-Canada will address the question specifically in cardiac surgery patients, where the risk of POD is especially high ^2– 9^.

**Table 1.** ENGAGES-Canada: Conceptualization – Intervention, Mediators, Outcomes, and Adverse Events. Assessment acronyms: EEG – Electroencephalogram; ICU – Intensive Care Unit.

ENGAGES-Canada: Conceptualization	
Intervention	· **EEG-guided reduction of anesthesia**
Mediators • Hypothetically linked both to the intervention as well as to the outcomes and the adverse events	·Intraoperative mediators ○Decrease in **EEG suppression** ○Decrease in anesthetic exposure ○Decrease in hypotension
Primary Outcome	· **Incident postoperative delirium**
Exploratory Secondary Outcomes	·Length hospital and ICU of stay
Other Exploratory Outcomes	·Postoperative **falls** incidence and association between delirium and falls ·Delirium duration and severity ·Postoperative **quality of life** ·Perioperative distress and dissociation ·Other delirium outcomes: delirium on day 0, time to delirium onset, sensitivity analyses, covariate adjustment · intraoperative mediating events: anesthetic concentrations, electroencephalogram suppression time and hypotension duration ·Functionality ·Cognition
Major Perioperative Adverse Events	·Undesirable movement ·Awareness with recall ·Serious complications (examples: major blood loss and transfusions, stroke, sternal wound infection, sepsis, dialysis, prolonged intubation) ·Death

Thus, the objective of the ENGAGES-Canada trial is to address whether reducing anesthetic administration based on EEG information during cardiac surgery primarily decreases the incidence of POD, and secondarily decreases the duration and severity of POD. Other relevant measures in the trial will be clinically relevant outcomes determined at the index hospitalization, at approximately 30-day postoperatively, and at approximately 1-year postoperatively.

## Methods

### Overview of the research design and study subjects

The Research Ethics Boards of the Montreal Heart Institute (2017-2164), the University of Manitoba (HS18290), Kingston University (ANAE-298-16), and the University of Toronto (17-5933) all approved the study. The details of the design of the ENGAGES-Canada study are included in this protocol and it contains the complete list of elements from the SPIRIT (Standard Protocol Items: Recommendations for Interventional Trials) checklist ^29, 30^. This study was registered with ClinicalTrials.gov ( NCT02692300) on 26 February 2016.

 The ENGAGES-Canada will randomize 1200 patients of 60 years old and older in a clinical trial designed to be pragmatic. The first patient randomized in the trial was enrolled in November 2016. Competent patients who provide informed consent and who are undergoing elective cardiac surgery with cardiopulmonary bypass are eligible to be included in the study. There are no absolute contraindications to EEG monitoring, and the design of the ENGAGES-Canada trial is largely pragmatic. Exclusion criteria include positive preoperative delirium screening. In addition, patients with hearing impairment, and those who are blind, illiterate, or do not speak French or English will be excluded because they will not be able to participate adequately in delirium screening. Finally, patients who have previously had general anesthesia and who have reported experiencing intraoperative awareness will also be excluded ^31^.

Participants are randomized to either one of two groups: in the guided group, anesthetic administration is guided largely by EEG waveform; in the other group, patients receive current usual anesthetic care for cardiac surgery. Delirium and other assessments take place preoperatively, and delirium is assessed postoperatively from day one to five while the patient is in the hospital. The primary outcome will be the incidence of postoperative delirium. As secondary and other outcomes, we will also examine stress reaction and dissociation at discharge as potential factors associated with delirium and predictors of postoperative psychiatric symptoms. At 30-days and at 1-year, follow-up assessments include the quality of life as related to health, cognitive functioning, and information on psychiatric symptoms and on the incidence of falls – collected via self-report.

### Recruitment

All patients in the study are asked to sign an informed consent (see extended data ^32^). Subjects are recruited through the Pre-Anesthesia Clinics, and baseline questionnaires are given at that time if they agree to participate. Perioperative clinics are staffed with anesthesiologists and nurses specialized in perioperative medicine to evaluate the perioperative risks of each patient. Patients can also be enrolled prior to surgery in hospital wards during the preoperative visit.

Based on data from previous clinical trials including those focused exclusively on cardiac surgery ^2, 8^, we expect the study population to be largely representative of the typical 25% female to 75% male ratio and there is no stratification of randomization by sex. Older and more vulnerable patients are often under-represented in clinical research. The patients enrolled in the trial should constitute a broad representation of older adults undergoing cardiac surgery in Canada.

### Randomization and blinding

Computer-generated assignment at the patient level is used for randomization. Patients who are eligible, and have given informed consent, are randomized to either usual care or to the protocol for guided anesthesia according to the EEG. A one to one ratio of randomization of the patients between usual care and the protocol for EEG-guidance is used. Within the research team, members are trained to enroll participants and assign the randomization between the usual care or the protocol for EEG-guidance. Anesthesiologists and their team learn of the group assignment after the patient is brought into the operating room; an opaque, sequentially numbered, sealed envelope is opened to reveal the study group. The randomization sequence is generated by the data analysis center. The anesthesiologist and the team caring for the patient intraoperatively are not blinded to the group assignment. The patients and family members are not aware of the group assignment. The research team members who are in charge of the assessment of delirium postoperatively remain blinded to the group assignment. Chart reviews are conducted by individuals blinded to the group assignment and blinded to the outcome of the postoperative delirium assessments ^29, 30^. Predictability of the randomization sequence is minimized by recording in a separate document, unavailable to the members of the research team who enroll participants and assign groups, the details of patients who have already been randomized.

### Protocol for EEG-guided anesthesia

Patients randomized to the EEG-guided group undergo anesthesia based on a pragmatic protocol to guide anesthesia administration according to EEG information. Prior to study participation, anesthesiologists are trained to recognize the typical EEG patterns of the different levels of anesthesia seen during anesthesia with volatile anesthetic agents; from the pattern in awake patients to the pattern typically found during general anesthesia. Practitioners are also encouraged to complete the online training provided on the website www.anesthesiaEEG.com as well as to watch two educational modules on the website www.icetap.org: one on waveforms of EEG and depth of anesthesia, and the other on Clinical decision making in anesthesia using the EEG. For sites using BIS processed electroencephalogram monitors, the proprietary processed BIS value is used in the EEG-guided group for the trial, as well as the raw waveforms of the EEG and all the numerical values seen on the monitor that are non-proprietary. These include the spectral edge frequency and the burst suppression ratio. For sites using a SedLine ^**®**^ monitor, the processed Patient State Index (PSi) in the EEG-guided group is used as well as the raw EEG waveform, spectrograms and suppression ratio. For sites using the NeuroSENSE ^**®**^ monitor, the processed WAVcns in the EEG-guided group is used as well as the raw EEG waveform, spectrograms and suppression ratio. To see clearly the low frequency slow delta waves, the EEG filter is turned off. If there is too much artifact in the EEG signal, practitioners may intermittently turn on the EEG filter to improve the resolution of the EEG trace. The anesthesiologists and their team are advised to inspect the waveform of the EEG to detect when there are periods of EEG suppression. EEG suppression can usually be recognized easily, and this pattern represents the main trigger, according to the protocol, for decreasing anesthetic administration. The low alarm is set at a value of 40 on the BIS monitor and NeuroSENSE ^**®**^ monitor or 25 on the SedLine ^**®**^ monitor. The sounding of this low alarm indicates an increased risk of a burst suppression pattern on the EEG (see PSI 25-50 white paper) ^33^. EEG index values less than manufactures’ recommendations (BIS and WAVcns values less than 40 or a PSi less than 25) indicate a second trigger for decreasing the administration of anesthetic agents. It is important to remember that the protocol of guidance of anesthesia according to the EEG waveform is suggestive and not prescriptive. Clinical judgment can inform deviations from the protocol according to the specific situations encountered. For all patients, regardless of the allocation group, the low volatile alarm (0.3 minimum alveolar concentration or clinician’s discretion) sounds to decrease the risk of intraoperative awareness. In the usual care group, the EEG monitor values are masked from the anesthesia team, except for the signal quality measures, and the data are stored for analysis of epochs of EEG suppression. For sites using the BIS monitors and NeuroSENSE ^**®**^ monitors, anesthesiologists see the signal quality index (SQI) only and for sites using the SedLine ^**®**^ monitor practitioners only see the impedance quality of the electrodes.

### Anesthesiologists fidelity to the EEG-guided anesthetic protocol

Monitoring devices can only influence and change clinical practice if important and useful information can be used by clinicians to make decisions regarding patient treatment during a case. Motivation to make decisions and act upon data from a monitoring device relies on familiarity with the device as well as evidence relating to outcomes. The use of EEG guidance of anesthesia in clinical practice is limited by the fact that electroencephalography is not formally part of the current residency curriculum. It is therefore not surprising that EEG guidance of anesthesia has not been adopted into usual anesthetic care. Focused training sessions have shown that anesthesiologists can readily learn the changes in EEG waveform associated with sedation and general anesthesia ^34^. Clinicians are capable of integrating the information from the EEG waveform when provided with clinical context and of estimating the BIS values associated with specific EEG waveforms ^34^. Arising from the results of this study, an international non-profit initiative focused on EEG education was launched: **I**nternational **C**onsortium for **E**lectroencephalograph **T**raining of **A**nesthesia **P**ractitioners.

Teaching the EEG waveform to anesthesiologists at our institutions and emphasizing the role of EEG in the guidance of anesthetic management are crucial ingredients to the success of the ENGAGES-Canada study. EEG derived parameters are captured electronically. This includes both the proprietary values, such as the BIS, WAVcns and PSi values, and non-proprietary values such as the suppression ratio. As a proof of concept, we were able to show in the first 102 patients enrolled to the ENGAGES-Canada trial that there was a reduction in both cumulative EEG suppression time and volatile anesthetic administration in the patients randomized to EEG-guided anesthetic care. Since our fundamental hypothesis for the ENGAGES-Canada study is that, in real-world practice, alterations in the management of anesthesia guided by the EEG waveform can prevent postoperative delirium, a crucial step was to confirm the ability of cardiac anesthesiologists to decrease the duration of EEG suppression by following the EEG-guided protocol.

### Outcomes

The primary outcome is the occurrence of incident delirium on postoperative days 1-5. Secondary outcomes include: length of ICU and hospital stay; duration and severity of delirium POD 1-5; incidence of falls and association between delirium and falls at 30 days and at 1 year; association between delirium and quality of life by PROMIS Global Health; predictors of Post-Traumatic Stress Disorder (PTSD) by PDEQ at 5 days and 30 days postoperatively; and predictors of Post-Traumatic Stress Disorder (PTSD) by PDEQ at 5 days and 30 days postoperatively. Exploratory outcomes include co-variates of delirium, functionality, and cognitive impairment at 30 days and at 1 year postoperatively. Pre-specified exploratory analyses include intraoperative mediating events such as anesthetic concentrations, electroencephalogram suppression time, and hypotension duration. Perioperative adverse events include undesirable intraoperative movement, awareness with recall, complications such as major blood loss and transfusions, stroke, sternal wound infection, sepsis, dialysis, prolonged intubation, and mortality rates at 30-day and at 1-year.

## Data collection

### Preoperative

Preoperative assessments are performed in the Pre-Anesthesia Clinic or hospital ward. These include demographic information and medical history, assessment of preoperative quality of life, psychiatric symptoms (i.e., anxiety, depression, alcohol use, trauma history, and posttraumatic stress disorder), objective and subjective cognitive functioning measures ^35, 36^, and evaluation of falls’ history. The purpose of the preoperative assessments is to identify cognitive and psychiatric disorders in order to better understand risk factors for postoperative delirium and its sequelae. For example, postoperative delirium has been associated with impaired performance on preoperative cognitive tests ^37^, and postoperative delirium predicts persistent postoperative cognitive decline ^6^. These known risk factors will be included in covariate analysis for the primary outcome and will be used in exploratory analyses. The following measures were selected for the assessment of cognitive and psychiatric symptoms based on their brevity, frequency of use in medical practice, validity for older adults, and lack of copyright restrictions: (1) 8-item AD8 Dementia Screen ^38^, (2) History of Delirium Screen, (3) Short Blessed Test ^39^, (4) 3-item Alcohol Use Disorders Identification Test (AUDIT-C), (5) 1-item surgical anxiety visual analogue scale, (6) Trauma History Screen, (7) Posttraumatic Stress Disorder Checklist (PCL-5; past-month PTSD assessment), (8) 4-item Primary Care PTSD Screen (PC-PTSD; lifetime PTSD assessment), (9) 4-item Patient Health Questionnaire (PHQ-4; anxiety and depressive symptoms), (10) 10-item Patient Reported Outcomes Measurement Information System (PROMIS) Global Health (physical and mental health related quality of life), (11) 4-item PROMIS Applied Cognition-Cognitive Concerns, (12) 4-item PROMIS Applied Cognition-Abilities (see extended data ^32^). Patients will also receive a questionnaire regarding history of falls. Baseline screening, informed consent, delirium assessments (described below), and self-report symptom measures take approximately 30–40 minutes. Research assistants obtain informed consent and administer AD8 Dementia Screen, History of Delirium Screen, Confusion Assessment Method (CAM long form; detailed below), and the Short Blessed Test; the remaining surveys can be completed independently if the patient has the capacity to do so. A script is provided to research assistants to introduce the psychiatric symptom measures (extended data ^32^). If patients request a direct referral for mental health related issues, they are referred to the appropriate clinician by the attending anesthesiologist in the Pre-Anesthesia Clinic.

### Perioperative period (up until discharge)

Delirium and pain are assessed postoperatively in the afternoon/evening while patients are in the hospital. Daily delirium assessments collected specifically for the ENGAGES-Canada study are entered into the Washington University School of Medicine Research Electronic Data Capture (REDCap) ^40^ database. EEG data, including BIS values, WAVcns, PSi values, and duration of EEG burst suppression are collected in both the guided as well as in the usual care group. In the usual care group, anesthesiologists are blinded to EEG data ^23, 41^. Perioperative data, including the repeated measures data, are saved for analysis from the intraoperative electronic data capture. As part of the routine data collection, we obtain for each patient a detailed medical history, surgical history, specific risk factors, medications, Blessed Dementia rating scale, screening for sleep apnea, laboratory values, medications during surgery, physiological readings, and parameters from the postoperative recovery period. At the time of discharge, patients will also complete a measure of immediate stress reactions (13-item Peritraumatic Distress Inventory; PDI) and peritraumatic dissociation (10-item Peritraumatic Dissociative Experiences Questionnaire; PDEQ) in the perioperative period, which have been found to be strong predictors of incident psychiatric disorders immediately following the stressful experience. Furthermore, they will complete an adapted 4-item PHQ-4 concerning more general anxiety and depressive symptoms, specifically during the perioperative period. These tests take patients approximately 5 minutes in addition to their CAM long form assessment (10 minutes).

### Postoperative delirium

The primary outcome of the study is the incidence of postoperative delirium. Assessment of postoperative delirium is conducted in patients that can be sufficiently aroused according to a Richmond Agitation and Sedation Score ≥ -3. Assessment of patients for delirium is performed from day one to day 5, once daily, in the afternoon/evening. A diagnosis of delirium for each patient is based on an approach combining a standardized daily assessment with a structured chart review. Members of the research team who are blinded to the treatment arm of the study assess patients for delirium using the Confusion Assessment Method (CAM long form) ^42^, or the Confusion Assessment Method for the Intensive Care Unit (CAM-ICU) ^43, 44^ for patients who are unable to speak (e.g., have a tracheal tube or tracheostomy). The CAM long form is deemed a viable tool that can be used by non-psychiatrists and by non-mental health professionals for delirium detection ^42^. The CAM long form has subsequently been validated in a number of studies and against a reference standard, with a sensitivity of >94% and a specificity of >89% ^45^. The CAM long form and the CAM-ICU are reliable instruments in good agreement with the DSM-IV criteria for delirium ^44, 46, 47^. To complete the delirium assessment of patients, a trained clinical researcher blinded to the CAM results and group assignment conducts a structured chart reviews for delirium detection. A positive CAM long form, or CAM-ICU, or chart review delirium detection ^48, 49^ is deemed sufficient and necessary to diagnose incident delirium in the ENGAGES-Canada trial. The approach combining a CAM long form interview, or a CAM-ICU, plus a chart review results in an increase in the sensitivity while retaining specificity in detecting incident delirium ^48, 49^. In addition, use of a chart review bolsters the pragmatic aspects of this trial, since it obtains information from a readily available source. Determination of delirium severity is based on the CAM-S long form measure or the CAM-ICU-7 Delirium severity instrument ^50, 51^.

The CAM long form assessment that is used in the trial was developed by Dr. Inouye and colleagues ^42^, and includes a brief cognitive battery. All study team members who perform delirium assessments undergo a structured training process. For the initial training, representatives from the research team participated in a full-day training program led by Dr. Inouye, the original creator of the CAM long form ^52^. Those who attended this initial training are overseeing the training of other team members. Trainees must demonstrate competence at both conducting CAM long form and CAM-ICU interviews and in scoring these interviews. To establish their ability to score CAM long form interviews, trainees accompany trained team members to conduct CAM long form interviews. A trained member of the research team conducts each CAM long form interview for patients enrolled in the trial. The trainee observes the interview, but scores the CAM long form independently. The trainee must agree with the trainer on the presence or absence of all 12 cognitive features assessed by the CAM long form on a minimum of two delirious and two non-delirious patients. After meeting the stipulations of training, the newly trained team member will conduct their first interview of a patient enrolled into the trial in the presence of a previously trained team member. Independently of the training, all team members who are participating in CAM long form assessments must view and rate nine videos of standard interviews of actors depicting delirious and non-delirious patients. Research team members must be trained on CAM long form assessments prior to completing any structured delirium chart reviews.

This structured process is employed to evaluate and confirm the quality of the assessments of delirium. In all cases the assessments of delirium are reviewed within three days by a fellow member of the research team to evaluate internal consistency of scoring and completeness. Once a month, all challenging delirium assessments are discussed in a conference call including investigators from all sites. Whenever there is ongoing disagreement regarding a CAM long form, CAM-ICU or delirium chart review, these are allocated to experts for adjudication (involving ES, SI). Ongoing interrater-reliability assessments of all study staff are planned annually. A random selection of CAM long form, CAM-ICU, and structured chart reviews are sent to experts for quality control purposes.

### 30-days postoperative

Self-report psychiatric and cognitive symptom measures are either mailed to patients 30-days post-surgery with a stamped and addressed return envelope, sent by e-mail, or obtained through a telephone call. The following measures are re-administered: Falls Questionnaire, AUDIT-C, PDEQ, PCL-5, PHQ-4, PROMIS Global Health, PROMIS Applied Cognition-General Concerns, and PROMIS Applied Cognition-Cognitive Abilities. These tests take approximately 15 minutes for patients to complete.

### 1-year postoperative

Self-report psychiatric and cognitive symptom measures are either mailed to patients 1-year post-surgery with a stamped and addressed return envelope, sent by e-mail, or obtained through a telephone call. The following measures are re-administered: Falls Questionnaire, AUDIT-C, PCL-5, PHQ-4, PROMIS Global Health, PROMIS Applied Cognition-General Concerns, and PROMIS Applied Cognition-Cognitive Abilities. Additionally, patients are contacted via phone and receive the Short Blessed Test. These tests take patients approximately 15 minutes to complete by writing. The phone call for the Short Blessed Test lasts approximately 3 minutes.

### Sample size calculation statistical analyses

Based on previous studies in cardiac surgery patients, we conservatively estimated a delirium incidence in the control group of 25% ^2– 9^ and based on meta-analyses of randomized trials we estimated a relative reduction in delirium with guidance of anesthesia based on EEG waveform of more than one-third ^3, 7, 24^. Assuming a two-sided alpha <0.05 and 1200 patients, we estimated >90% power to be able to detect an absolute decrease in the incidence of delirium of 8% ^27^. With a more stringent two-sided alpha <0.005, we estimated >70% power. We anticipate that most patients will be available for primary outcome ascertainment (i.e. incident postoperative delirium assessment). Continuous variables will be presented as mean (SD) or median (interquartile range [IQR]), depending on their distributions. Chi-square or Fisher’s exact tests will be used for comparisons of discrete data. Unpaired Student’s t or Mann–Whitney U tests will be used for comparisons of continuous data, depending on their distributions. Confidence intervals for median differences will be calculated using Hodges-Lehmann estimates, and for differences between proportions we will use Newcombe’s method with continuity correction ^53^. For the primary outcome, the proportion of patients with incident postoperative delirium will be compared between the two study groups. We will conduct three post-hoc sensitivity analyses to assess whether improved clinician fidelity to the guided protocol might alter the primary outcome. Within the guided group 25% of cases will be excluded with the (1) longest cumulative electroencephalogram suppression time; (2) longest cumulative time with BIS, WAVcns <40 or PSi <25; and (3) highest median volatile anesthetic concentrations. To compare time to delirium onset between groups, we will construct Kaplan-Meier curves for each group and conduct a log-rank test. We will perform a covariate adjustment including likely risk factors for delirium (including age, sex, history of depression, history of delirium, AD* Test, Short Blessed Test, comorbidity index, and study center) using two methods: (1) logistic regression and (2) standardized estimator combined with bootstrapped 95% confidence intervals (CIs) ^54^. We will compare delirium incidence between groups, segregated by Charlson Comorbidity Index categories ^55^. Patients will be assessed based on their randomization group in accordance with an intention-to-treat approach. We will also conduct a per protocol sensitivity analysis: when clinicians in the usual care group view EEG data, these patients will be included in the guided group; and where there are technical difficulties in viewing electroencephalogram data in the guided group, these patients will be included in the blinded group. Patients who cannot be assessed for delirium will be excluded from the primary outcome analysis. In two sensitivity analyses, these patients (unless they die during surgery) will either all be assumed to have had incident delirium or not to have had incident delirium. We will compare preoperative characteristics between respondents and alive non-respondents to 30-day postoperative surveys. We will do the same for the 1-year postoperative surveys. Results will be presented with 95% CIs. All significance testing will be two-sided, with P values <0.05 considered as providing suggestive evidence and P values <0.005 as providing more compelling evidence ^56^. The statistical analyses will be performed with SAS, V.9.4 (SAS Institute, Cary, North Carolina) and/or STATA, V.14.2 (StataCorp LP, College Station, Texas).

### Secondary outcomes (falls, quality of life, psychiatric status, cognitive functioning)

Estimations of the primary outcome (incidence of delirium) was used for the calculation of the overall sample size of our study. For secondary analyses, it is assumed that 80% (∼1000 patients) of the trial population will have completed the 30-day and 1-year follow-up survey. With a sample size of 1000 patients, it should be possible to detect a difference of 0.5 points (SD of 2.5) in mean score of the Physical Health Score from baseline to 30-day and 1-year follow-up survey between the usual care and guided group with a power of >80% and a 2-sided alpha level of p<0.05. Secondary outcomes and exploratory analyses will be examined in a number of ways; including multivariable regressions.

### Strengths of the study and limitations

***Strengths.*** Several important strengths can be ascribed to the ENGAGES-Canada study. The clinical trial is randomized, pragmatic, includes high volume sites, and is conducted in real life settings. The interventions to avoid EEG suppression are easily implemented and the primary outcome, the incidence of delirium, is of tremendous importance to patients, health care systems and society. In relation to the primary outcome (delirium incidence), the CAM instrument has been validated against reference standard DSM-IV and DSM-IV-TR criteria in multiple studies, and has been appraised as one of the two most reliable instruments for detecting delirium in a research context ^57^. The CAM long form has been demonstrated to have excellent psychometric properties for both hypoactive and hyperactive delirium ^58^. Programmatic training, coupled with the highly structured use of the CAM long form (as we are doing in the trial), has demonstrated excellent inter-rater reliability of researchers ^28, 59^. In addition, by complementing the CAM long form (or CAM-ICU) with validated chart review ^49^, we are bolstering delirium detection and minimizing missing primary outcome assessments. Since many components of the study are already incorporated into existing processes and infrastructures at participating Canadian sites, its implementation can be efficient. Enrollment is easily achieved within the flow of the clinic. The study is largely conducted by anesthesiologists and is integrated into the course of their clinical workday. Randomization is implemented in the operating room at the point of patient care because the anesthesia protocols do not require any advanced preparation or lead-in time. Enrollment of vulnerable patients 60 years old and older allows us to study a population that is understudied in clinical research. The importance of studying and understanding this targeted population lies in the potential of significant benefits from reducing the incidence of postoperative delirium and its related outcomes. Secondary outcomes such as patient-reported health-related quality of life factors are especially relevant to older patients who remain a largely understudied population. Trial feasibility is enhanced by the participation of investigators from multiple disciplines, who as a group has managed to establish a track record of successful collaboration and the completion of several major clinical trials (Canadian PACT group ^60– 62^). Since EEG guidance of anesthesia is straightforward and inexpensive, positive results from the study could provide strong effectiveness evidence, and would facilitate implementation, sustainability and dissemination of the EEG-guided protocol on a national level in Canada, as well as in other countries.

***Limitations.*** Some limitations for the trial should be considered. The EEG-guided protocol can only be effective if anesthesiologists adhere to it. Including patients in a clinical trial aiming at the prevention of the incidence of postoperative delirium and providing patients and family members with educational material on the subject could possibly decrease the incidence of postoperative delirium. Three methods of assessment will be used to diagnose postoperative delirium, a pragmatic structured chart review, the full CAM and the CAM-ICU. The CAM-ICU has a decreased sensitivity compared to the CAM long form. However, the CAM-ICU is broadly used and has been validated for patients who are unable to speak (i.e., with a tracheostomy or a breathing tube in place). Available data from participating centers indicate that the vast majority of patients enrolled in the in the study will be extubated within the first 48 hours. As a result, delirium assessment will usually be obtained with the more specific and sensitive instrument – the CAM long form. Since the 30-day and the 1-year follow-ups for patient are based on self-report outcome measures, a potential limitation is an incomplete follow-up at these periods in time. Consequently, a sensitivity analysis was performed in our power calculations to take into account this possible attrition (80% response rate at 30-day and at 1-year). We plan to improve follow-up by contacting patients first by mail and, if unsuccessful, by e mail or telephone calls. Postoperative delirium could be missed with recurrent assessments because it is well known to be a fluctuating disorder. In our efforts to minimize missed diagnosis of postoperative delirium, we have included a structured chart review. This validated complementary approach has been shown to increase the detection of postoperative delirium ^48, 49^.

### Potential risks, alternatives and benefits

***Benefits.*** If our hypotheses are confirmed, patients randomized into the group with the EEG-guided anesthesia will have a greater chance of avoiding postoperative delirium and its consequences’ downstream; including decrement in quality of life, falls, and cognitive decline. Evaluation of any potential unanticipated negative effects of EEG-guidance by investigating this range of psychiatric, cognitive, and functioning outcomes will also be possible.

***Risks.*** There are few risks associated with this study. Breach of confidentiality is a rare risk. Psychiatric symptom self-report measures may also cause patients temporary distress, but it may also provide the opportunity for distressed patients to obtain community mental health resources and/or appropriate referral. EEG guidance of anesthesia poses a theoretical risk of intraoperative movement and/or awareness with recall. Nevertheless, previous studies have not found an increased incidence of awareness with randomization of patients to protocols using EEG guidance of anesthesia ^14, 15, 63^. In our study, intraoperative awareness with recall will be tracked within 48 hours of extubation postoperatively with a modified Brice interview ^64^ (see extended data ^32^). Review of adverse events by a data-safety monitoring committee and the PI, in consultation with the institutional review board, might lead to the recommendation to stop the trial in the event of increased reporting of intraoperative awareness in the EEG-guided group. In the informed consent for this study, patients are made aware of the rare risk of intraoperative awareness with recall. Serious side effects will be reported to the Ethics Committees and to the study’s data safety monitoring board. There is no financial compensation for the participating patients and all expenses related to the study are incurred by the participating research sites.

### Minimizing risks and confidentiality

Members of the research team will share protected and necessary health information. Confidentiality will be protected by storing charts in locked cabinets located inside locked research offices. Demographic information and electronic data will be password-protected on an electronic database and will be stored on the network drive of the participating sites. Computers accessing this network will also be password-protected. This information will be entered by a designated member of the research team. The documents and data used for evaluation or statistical analyses will only contain coded numbers. Patients are free to decline participation in the study without penalties for the care they receive. Data management and processing will be done by the Division of Biostatistics Informatics Core at Washington University. Washington University is part of a consortium of institutional partners working to provide clinical trial data and management of research through a software toolset and workflow methodology for electronic collection. REDCap (Research Electronic Data Capture) data collection projects rely on a thorough study-specific data dictionary defined in an iterative self-documenting process by all members of the research team with planning assistance from the Division of Biostatistics Informatics Core. A well-planned data collection strategy for individual studies results from an iterative development and testing process. REDCap servers are securely housed in an on-site limited access data center managed by the Division of Biostatistics at Washington University. All web-based information transmission is encrypted. The data is stored on a private network, firewall-protected. Individual user identifiers and passwords are given to all users with restricted access depending on the specific role in the study. REDCap was developed specifically around HIPAA-Security guidelines, and is implemented and maintained according to University guidelines. More than 500 academic and/or non-profit consortium are supported by REDCap on 6 continents and 38,800 research end-users.

### Safety monitoring and adverse event reporting

Adverse events during the study will be monitored by the research team. If a serious adverse event (SAE) occurs, it will be directly reported to the REB in accordance with REB stipulations. For this pragmatic study, the safety monitoring plan seems appropriate. We conducted three large clinical studies where EEG-guided general anesthesia was given to half of the 28,000 patients studied. From these studies, no adverse events were attributable to EEG-guided anesthesia ^14, 15, 63^. There is therefore no reason to think that adverse events attributable to EEG-guided anesthesia would occur in the present study. Since this is a low risk trial, the study has an appropriate data and safety-monitoring plan. The Data Safety Monitoring Board (DSMB) provides oversight of the ENGAGES-Canada Clinical Trial. The DSMB will examine data for the safety of the participants and will review the general conduct of the study ^65^. Recommendations will be made by the DSMB regarding the modification, continuation, or termination of the study ^66^. The expertise of the DSMB members will allow examination the cumulative data, protection of the integrity of the clinical experiments to which the patients have consented to participate, and to assure the regulatory bodies and the public that conflicts of interest do not compromise either patient safety or trial integrity ^67^. An annual review of the safety events will be conducted by the DSMB. The DSMB may advise to stop the trial for safety concerns, but not for reasons of efficacy or because of futility ^65^. Implausibly large treatment effects are found in trials that stop early for benefit, especially with small number of events ^68^. Trials that have terminated early show greater effect sizes compared to trials that are continued, independent of existing statistical rules for terminating the trial ^69^.

***Early stoppage of the trial.*** On average, we can assume that patients in the group with EEG-guided anesthesia will receive, on average, lower concentrations of inhaled anesthetic agents during surgery. Studies using similar strategies have been reported without increased incidents related to intraoperative awareness with recall ^70, 71^. Nevertheless, we cannot exclude the possibility that patients receiving lower concentrations of inhaled anesthetic agents could experience a higher incidence of intraoperative awareness. Therefore, it will be necessary to compare the incidence of intraoperative awareness between groups in the trial. We recommend to the DSMB that this occurs at mid-point during the study (600 patients). Results from a one-tailed comparison of the incidences of awareness between the groups showing an increased incidence of awareness in the EEG-guided group (with a p value <0.05) will result in consideration by the DSMB to terminate the study. The severity of the awareness experiences; including reports of pain, paralysis, and distress could be taken into consideration by the DSMB ^72^. Furthermore, other measures of peritraumatic distress and dissociation are administered at discharge, which will further allow for an evaluation of severity of awareness. Besides intraoperative awareness, a lower administration of inhaled anesthetic agents is not hypothesized to result in adverse outcomes that would be clinically important such as death, myocardial infarction, and/or stroke. Other events that could be associated with decreased anesthetic administration include intraoperative patient movement and higher intraoperative blood pressure and/or heart rate. These measures have unclear clinical relevance if not related to more severe adverse events and, as such, should not influence the decision to stop the study early.

It is recommended that an interim efficacy analysis of EEG guidance should not be performed by the DSMB with respect to the primary outcome of incident delirium. Some data support the hypothesis that EEG-guided anesthesia decreases the incidence of postoperative delirium, but other data do not support this hypothesis ^7, 71, 73, 74^. A higher incidence of postoperative delirium in the EEG-guided group would conflict with the results of current studies. The results of a partially completed trial would therefore be unlikely to provide compelling evidence to influence future clinical practice for anesthesia guided by EEG waveform in this patient population.

### Ethics and dissemination

All major changes to the protocol will be decided by the trial steering committee. The committee will communicate these changes to the REB and appropriate parties.

The final dataset of the trial is the property of the investigative team and shall not be shared without permission from the principal investigator. Dissemination plans include presentations at local, national and international scientific conferences. Every effort will be made to publish results of the ENGAGES-Canada trial in a peer-reviewed journal. Dissemination of results to study participants and their family members will be available upon request. Updates and results of the study will be available to the public at www.clinicaltrials.gov.

### Pre-specified additional analyses and sub-studies

Canadian centers participating in the ENGAGES-Canada Trial are encouraged to develop sub-studies related to the trial if approved by their local REBs. No sub-study can be published prior to pre-approval by the ENGAGES-Canada Trial Management Committee.

a) *Association between delirium and clinically relevant outcomes*
  The Engages-Canada trial will explore the associations between outcomes that are clinically relevant such as cognitive decline, falls, functional decline, ICU and hospital length of stay, and mortality; and the severity, incidence, and duration of delirium.
b) *Differences between observational and patient-reported pain scores*
  Patients with postoperative delirium may lack the ability to express verbally the level of pain they are suffering ^75^. Understanding that uncontrolled postoperative pain may be related to delirium emphasizes the importance of the assessment and treatment of postoperative pain. A comparison of behavioral pain assessment and patient reported pain will be made in delirious and non-delirious patients ^75^. These pain scores can also be explored in the context of psychiatric status as high comorbidity rates exist.
c) *Patient outcomes and delirium*
  The association between cognitive status, falls, and quality of life with postoperative delirium will be evaluated at 30-day and at 1-year post surgery.
d) *Anesthesia depth and postoperative outcomes*
  Secondary measures, that can be related to the difference in anesthesia depth between the two groups of patients, will be evaluated since patients in the EEG-guided group will almost certainly be exposed to lower concentrations of anesthetic agents. We will then be able to compare our results with an ongoing randomized clinical trial looking at the effects of depth of anesthesia on a range of outcomes ^76^, such as cardiac arrest, pulmonary embolus, death, stroke, myocardial infarction, cardiac arrest, surgical site infection, ICU and hospital length of stay, and intraoperative awareness.
e) *Post-traumatic stress disorder (PTSD) outcomes*
  There is growing interest in understanding PTSD post-operatively given its high prevalence in surgical cohorts ^77^. The current study allows for an incidence investigation, as a history of PTSD is assessed at baseline. This study will allow for a thorough investigation using contemporary DSM-5 PTSD criteria. Incident PTSD at 30-day and at 1-year follow-up will be assessed and several potential mechanisms can be explored including: pre-operative factors, dissociation and distress during the surgical period, delirium, surgical complications, psychiatric and cognitive status, and depth of anesthesia.
f) *Decreases in Regional Cerebral Oxygen Saturation and EEG Suppression Patterns*
  Some of the participating sites measure Regional Cerebral Oxygen Saturation (rSO2) on a regular basis. Several studies have shown an association between significant perioperative decreases in rSO2, cognitive decline, and postoperative complications ^61, 78, 79^. We therefore intend to explore the relationship between EEG suppression and cerebral desaturations.

## Study status

To date two interim analyses have been performed, one at 240 patients and another at 570 patients. While randomization remains blinded, both analyses confirmed separation of the groups in term of total time in EEG suppression pattern (control group with more suppression) as well as a total incidence of delirium within the range of the predicted value used for the original power analysis.

## Strengths

- The ENGAGES-Canada study is a multi-center clinical trial, conducted with a pragmatic design to reflect the clinical setting encountered in real-life.
- Cardiac surgery patients are at high risk for postoperative delirium.
- Postoperative delirium is important to patients, healthcare providers, and society.
- Guidance of anesthesia by electroencephalography is low-cost and could be broadly accepted.
- As a secondary aim, this study will also include cognitive and psychiatric outcomes 30-days and 1-year after surgery.

## Limitations

- The protocol-guiding anesthesia with electroencephalography can only be effective if anesthesiologists adhere to it.
- Delirium is a disorder that fluctuates in the course of 24 hours. It can be easily missed with scheduled assessments, regardless of the rigor of these assessments.
- When patients are unable to speak, delirium assessment might be less sensitive.
- Incomplete follow-up at 30 days and 1-year postoperatively might limit some of the interpretations.
- Self-reported cognitive and psychiatric symptom measures are less reliable than structured clinical interviews.

## Data Availability

No data is associated with this article. Open Science Framework. ‘Protocol for the electroencephalography guidance of anesthesia to alleviate geriatric syndromes (engages-Canada) study: A pragmatic, randomized clinical trial’, https://doi.org/10.17605/OSF.IO/DXY27 ^32^ This project contains the following extended data: ENGAGES-CANADA Protocol Appendix.pdf (PDF file containing all study questionnaires and screening materials) ENGAGES-CANADA Script.pdf (script for research assistants to introduce the psychiatric symptom measures) ENGAGES-CARDIAC Conscent French.pdf (Consent form, French) ENGAGES-CARDIAC Consent English.pdf (Consent form, English) Open Science Framework: SPIRIT 2013 checklist for ‘Protocol for the electroencephalography guidance of anesthesia to alleviate geriatric syndromes (engages-Canada) study: A pragmatic, randomized clinical trial’, https://doi.org/10.17605/OSF.IO/DXY27 ^32^ Data are available under the terms of the Creative Commons Zero “No rights reserved” data waiver (CC0 1.0 Public domain dedication).
