# Supplementary material for: Protocol for the electroencephalography guidance of anesthesia to alleviate geriatric syndromes (ENGAGES-Canada) study: A pragmatic, randomized clinical trial
Source: F1000Res. 2023 Jan 13;8:1165. Originally published 2019 Jul 23. [Version 2] doi: 10.12688/f1000research.19213.2 (PMC6760454; doi:10.12688/f1000research.19213.2)
Supplement: Peer review history

# Peer review: Reviewer response for version 1

**DOI:** 10.5256/f1000research.21053.r51528

**Published:** 2019-09-24

**Authors:** Lian Kah Ti

**Affiliations:** 1 Department of Anaesthesia, National University Health System (NUHS), Singapore, Singapore

This trial is a pragmatic trial modeled after the ENGAGES trial published in JAMA in 2019. The findings are of importance because it may validate the findings of the ENGAGES trial, which were opposite of what was expected based on several meta-analysis of previous data.  

 This trial also gives the authors an opportunity to address some of the criticisms of the ENGAGES trial. These include:

1. By concentrating on cardiac surgery, this removes the heterogeneity of surgeries of the original trial. 
2. Achieve a better separation of the time spent in burst suppression of the groups. This is likely to happen simply due to the passage of time, as anesthesiologists are more aware of the problem of delirium today, and are more comfortable using lower MACs of volatile agents.
3. Excluding the outliers in each group. 

However, there are several concerns, as below.

1. The use of different "black box" indices to guide MAC is based on the assumption that they are equal. However, being a pragmatic trial, it is perhaps a reflection of true practice.
2. The management of anesthesia during bypass is not clear, including the type of anesthesia (volatile vs. propofol), the involvement of the perfusionists, and the perfusion targets.
3. Assessment of delirium is still once a day. Because of the fluctuating course of delirium, the protocol is heavily reliant on chart reviews, which may underestimate the incidence.

Is the study design appropriate for the research question?

Yes

Is the rationale for, and objectives of, the study clearly described?

Yes

Are sufficient details of the methods provided to allow replication by others?

Yes

Are the datasets clearly presented in a useable and accessible format?

Not applicable

Reviewer Expertise:

Important clinical outcomes after major surgery

I confirm that I have read this submission and believe that I have an appropriate level of expertise to confirm that it is of an acceptable scientific standard.

# Peer review: Reviewer response for version 1

**DOI:** 10.5256/f1000research.21053.r52096

**Published:** 2019-08-30

**Authors:** Oluwaseun Johnson-Akeju

**Affiliations:** 1 Department of Anesthesia, Critical Care and Pain Medicine, Massachusetts General Hospital (MGH), Harvard Medical School (HMS), Boston, MA, USA

The protocol for the ENGAGES-Canada is well written and presented according to established guidelines. In brief, the protocol is constructed around the hypothesis that eliminating or reducing the incidence of burst suppression would decrease incident delirium. 

1. It may be beneficial to explicitly address whether the effects of cardiopulmonary bypass time, cardiopulmonary bypass temperature and total circulatory arrest would be addressed (in the context of burst suppression) in exploratory analyses.
2. It is reasonable to make clear how anesthetic management during cardiopulmonary bypass is managed. Do all centers use inhaled anesthetic-drugs and will the EEG be used to manage drug cardiopulmonary bypass drug dosing versus performed empirically by the perfusionist.
3. I missed whether/what anesthetic drugs are being collected intraoperatively for exploratory analyses (there is mention of anesthetic depth in the prespecified analysis - EEG?). Burst suppression may be a "non-modifiable readout" of deliriogenic brains. Nuanced exploratory analyses of anesthetic-drug/index values etc. may be helpful.

Is the study design appropriate for the research question?

Yes

Is the rationale for, and objectives of, the study clearly described?

Yes

Are sufficient details of the methods provided to allow replication by others?

Yes

Are the datasets clearly presented in a useable and accessible format?

Yes

Reviewer Expertise:

Anesthesiology

I confirm that I have read this submission and believe that I have an appropriate level of expertise to confirm that it is of an acceptable scientific standard.
